# Babies get it right

**DOI:** 10.7554/eLife.08232

**Published:** 2015-06-02

**Authors:** Hillary Hadley, Lisa Scott

**Affiliations:** Department of Psychological and Brain Sciences, University of Massachusetts Amherst, Amherst, United Stateshhadley@psych.umass.edu; Department of Psychological and Brain Sciences, University of Massachusetts Amherst, Amherst, United Stateslscott@psych.umass.edu

**Keywords:** face recognition, infants, right hemisphere, natural images, visual categorization, human

## Abstract

Infants use a region on the right side of their brain to distinguish between human faces and objects.

**Related research article** de Heering A, Rossion B. 2015. Rapid categorization of natural face images in the infant right hemisphere. *eLife* **4**:e06564. doi: 10.7554/eLife.06564

**Image** A selection of the images shown to infants to test their ability to recognize human faces

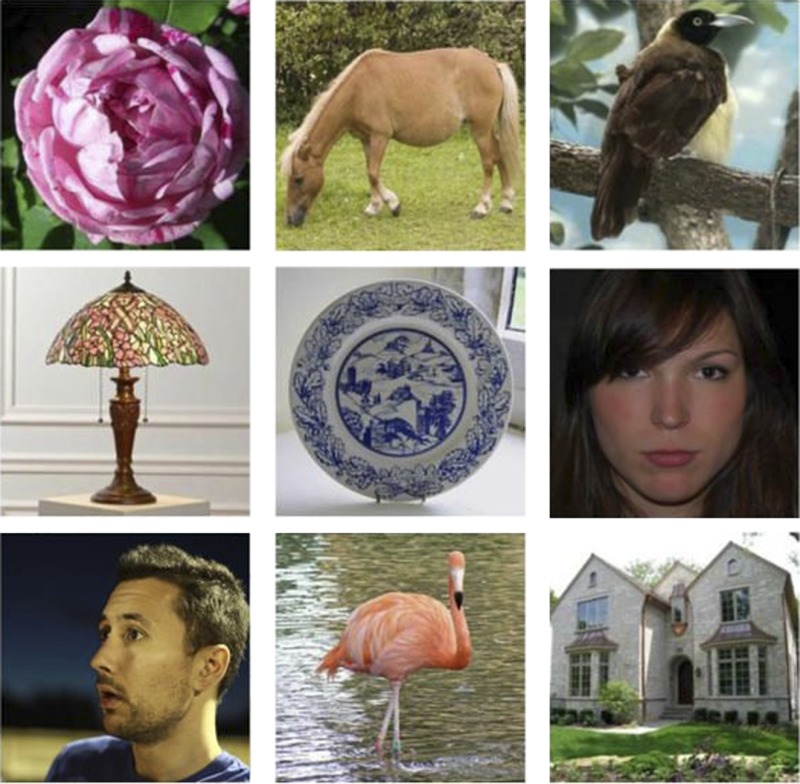

Human faces are an important part of social interactions. We use them to recognize a friend, to gauge someone's mood, or to figure out where to direct our attention. But before engaging in any of these activities, we must first identify a face as a face.

The fundamental question of how a perceptual system, such as the one underlying face recognition, becomes organized in the brain is important for understanding how changes in the brain lead to changes in behavior. Studying face perception in developing infants could help us to understand the parts of the brain that contribute to adult face perception. It might also reveal how face-processing abilities can be impaired in some populations, such as people with Autism.

In adults, the right hemisphere of the brain is critical for recognizing faces. Damage to the right hemisphere, but not the left hemisphere, can impair face recognition. Moreover, the right hemisphere produces larger brain responses than the left hemisphere when a face is seen. This has been witnessed using two different neuroimaging methods. The first, called functional magnetic resonance imaging (fMRI), measures the flow of blood around the brain and relates this to brain activity (e.g., Kanwisher et al., 1997). The second directly measures event-related potentials (ERPs)—the electrical response of a brain region to a stimulus (e.g., Rossion et al., 2003).

In children, there is also evidence that the right and left hemispheres of the brain respond differently to faces (Scherf et al., 2007). Recently it was reported that the response of the right hemisphere to faces is intricately linked to changes that occur in the left hemisphere when children learn to read (Dundas et al., 2014). To date, the majority of studies on infants (who are too young to read) have found no significant differences in how the two sides of the brain respond to faces (e.g., de Haan and Nelson, 1999; Gliga and Dehaene-Lambertz, 2007). However, one group did find response differences between hemispheres when comparing faces to markedly less complex stimuli (patterns of colored dots) (Tzourio-Mazoyer et al., 2002).

Now, in *eLife*, Adélaïde de Heering and Bruno Rossion from the University of Louvain have used a fast periodic visual stimulation (FPVS) approach to explore face perception in a group of infants aged between four and six months (de Heering and Rossion, 2015). This approach involves presenting images at a rapid, fixed rate in order to induce brain responses that occur at the same rate (often defined as ‘steady-state visual evoked potentials’, SSVEPs; Regan, 1966; for a review, see Norcia et al., 2015).

de Heering and Rossion report that, in the brains of these infants, faces are represented as a distinct category of objects, separate from other categories of objects such as plants or man-made objects. This distinction can be seen most prominently in the response recorded over the right occipito-temporal brain region, which is near the back of the brain. Importantly, faces that vary in size, viewpoint and features (such as the expression and the gender of the faces) are all categorized as faces. This is even the case when the images include naturalistic backgrounds.

These findings provide evidence that by the time they are six months old, infants possess a relatively robust ability to identify that faces are different from objects, and can do so in a realistic context. Moreover, the larger face-related brain responses recorded over the right hemisphere suggest that the right hemisphere of the brain has begun to preferentially respond to faces by six months of age. These findings also complement previous behavioral and ERP work suggesting that infants can distinguish between faces and objects in the first year of life (for a review, see Scott and Nelson, 2004).

Although this technique has been successfully used in infant studies of low-level vision (e.g., Braddick et al., 1986), de Heering and Rossion are among the first researchers to demonstrate the effectiveness of the FPVS technique using complex images in infant research. This is an important addition to the developmental scientist's toolbox and will greatly expand our ability to characterize brain development in infants even before they begin to talk. This technique has been successfully used in a variety of adult investigations but to our knowledge only one other published study reports results from this method with infants (Farzin et al., 2012).

The fact that the FPVS technique can be applied to infant populations has a number of benefits for researchers. Infants can be exposed to hundreds of trials and several conditions within minutes, and no verbal or motor response is required. This large amount of data, collected in a short period of time, results in a higher of proportion data suitable for analysis than in studies using behavior or standard ERP approaches. The increased number of trials also allows researchers to use a variety of visual stimuli that vary in shape, size and orientation, leading to conclusions that are more generalizeable and relevant to real-world situations. Relative to other methods, the FPVS method measures infant brain responses objectively, allowing for precise testing of predictions and easy comparisons across investigations. Finally, the FPVS method measures how the brain tells the difference between various stimulus conditions and provides a direct link between this response and the behavioral tasks commonly used to study infant perception, learning and memory.

## References

[bib1] Braddick OJ, Wattam-Bell J, Atkinson J (1986). Orientation-specific cortical responses develop in early infancy. Nature.

[bib2] de Haan M, Nelson CA (1999). Brain activity differentiates face and object processing in 6-month-old infants. Developmental Psychology.

[bib3] de Heering A, Rossion B (2015). Rapid categorization of natural face images in the infant right hemisphere. eLife.

[bib4] Dundas EM, Plaut DC, Behrmann M (2014). An ERP investigation of the co-development of hemispheric lateralization of face and word recognition. Neuropsychologia.

[bib5] Farzin F, Hou C, Norcia AM (2012). Piecing it together: infants' neural responses to face and object structure. Journal of Vision.

[bib6] Gliga T, Dehaene-Lambertz G (2007). Development of a view-invariant representation of the human head. Cognition.

[bib7] Kanwisher N, McDermott J, Chun MM (1997). The fusiform face area: a module in human extrastriate cortex specialized for face perception. Journal of Neuroscience.

[bib8] Norcia A, Appelbaum G, Ales J, Cottereau B, Rossion B (2015). The steady-state visual evoked potential in vision research: a review. Journal of Vision.

[bib9] Regan D (1966). An effect of stimulus colour on average steady-state potentials evoked in man. Nature.

[bib10] Rossion B, Joyce CA, Cottrell GW, Tarr MJ (2003). Early lateralization and orientation tuning for face, word, and object processing in the visual cortex. NeuroImage.

[bib11] Scherf KS, Behrmann M, Humphreys K, Luna B (2007). Visual category‐selectivity for faces, places and objects emerges along different developmental trajectories. Developmental Science.

[bib12] Scott LS, Nelson CA, Oldman JM, Riba MB (2004). The developmental neurobiology of face perception. Review of Psychiatry Series.

[bib13] Tzourio-Mazoyer N, De Schonen S, Crivello F, Reutter B, Aujard YB (2002). Neural correlates of woman face processing by 2-month-old infants. NeuroImage.

